# Survival Rate of Extremely Low Birth Weight Infants and Its Risk Factors: Case-Control Study in Japan

**DOI:** 10.1155/2013/873563

**Published:** 2013-11-25

**Authors:** Masaki Ogawa, Yoshio Matsuda, Eriko Kanda, Jun Konno, Minoru Mitani, Yasuo Makino, Hideo Matsui

**Affiliations:** ^1^Perinatal Medical Center, Tokyo Women's Medical University Hospital, Kawadacho 8-1, Shinjuku, Tokyo 1628666, Japan; ^2^Department of Obstetrics and Gynecology, Tokyo Women's Medical University, Tokyo 1628666, Japan

## Abstract

*Aim*. To clarify the effect of perinatal events on the survival of ELBW infants in Japan. *Methods*. 1,713 ELBW infants, from 92,630 live births in 2001 and 2002, born at 22–36 weeks of gestation were registered. Case was defined as death at discharge. The relevant variables were compared between the cases (*n* = 366) and the controls (*n* = 1,347). *Results*. The total survival rate was 78.6%. There was a significant difference between the survival rate in cesarean and vaginal delivery at 24–31 weeks of gestation. Cesarean delivery in infants with a birth weight >400 g was significantly advantageous to the survival rate of ELBW infants than vaginal delivery. The significant contributing factors were gestational age at delivery (OR: 0.97), Apgar score at 5 min (0.56), antenatal steroid (0.41), and birth weight (0.996). Nonvertex presentation (1.81), vaginal delivery (1.56), and placental abruption (2.50) were found to be significantly associated with neonatal death. *Conclusions*. Cesarean section might be advantageous for survival in ELBW infants over 24 gestational weeks or 400 grams of birth weight. Nonvertex presentation, vaginal delivery, and placental abruption could be significant risk factors for survival of ELBW infants.

## 1. Introduction

Prematurity is a particularly significant risk factor for survival of the neonate and is associated with increased perinatal mortality. The prognosis of low birth weight (LBW) infants has been improved drastically with advances in perinatal medicine. More than 80% of infants survive delivery at 24 weeks of gestation in many perinatal centers in Japan [1]. Interest has now turned to improvement of the intact survival rate of extremely low birth weight (ELBW) infants. To save the lives of ELBW infants, particularly those born at 22-23 gestational weeks, is thought to be at the frontier of current perinatal medicine. Gestational weeks 22 to 23 seem to be a limit of viability.

There are many perinatal factors that might influence the prognosis of ELBW infants. It has been well known that antenatal steroid administration improves the prognosis as well as survival [2], whereas vaginal delivery in nonvertex presentation is an adverse factor [3–5]. However, indication of cesarean section in extremely preterm infants less than 24 weeks is a matter of wide debate. This debate is also of great importance in Japan. To reach a conclusion, a nationwide survey in Japan is needed.

The World Health Report 2005 stated that the neonatal mortality rate within 28 days was 1.8 per 1000 live births in Japan, which was the next best data after Singapore (1.1 per 1000) [6]. It meant that medical care and treatment for neonates in Japan was the highest level in the world. Consequently, analysis of the risk factors for mortality in ELBW infants in Japan will shed light on critical problems found during the perinatal period.

The purpose of this study was to clarify the survival rates in ELBW infants in Japan, to reveal the effect of perinatal events on the survival of ELBW infants, and to identify risk factors associated with survival rates in perinatal centers in Japan.

## 2. Materials and Methods

### 2.1. Study Design and Data Source

The study protocol was reviewed and approved by the Ethics Committee of Tokyo Women's Medical University. Data were collected from 92,630 live births in 2001 and 2002 in the Japan Perinatal Registry Network database, which was managed by Japan Society of Obstetrics and Gynecology. It includes all live births and stillbirths at 125 medical centers in Japan in 2001 and at 133 medical centers in 2002, including almost all level III hospitals. We restricted our analysis to women who delivered a single live infant at 22 or more weeks.

The obstetric notes on the mothers were reviewed by a researcher unaware of the children's outcome. More than 20 variables were assessed, including demographic data and prenatal events. The pregestational factors were age, smoking habit, prenatal care, and the maternal medical history (complications, family history, and history of the relevant current pregnancy). The prenatal events were the presence of vascular disease, polyhydramnios, urogenital infection, and preeclampsia/superimposed preeclampsia. Details of the diagnosis, onset, duration, and clinical management of any relevant condition were also recorded.

### 2.2. Case Identification and Putative Risk Factor Selection

ELBW infant was defined as that born weighing less than 1,000 g, and very low birth weight (VLBW) infant was defined as less than 1,500 g. A total of 1,713 ELBW infants born at 22–36 weeks of gestation were registered in 2001 and 2002. Multiple pregnancy, chromosomal abnormalities, and phenotypical anomalies were excluded. Case was defined as death at discharge. The other cases, that is, survival at discharge, were used as controls. The relevant variables were compared between the cases (*n* = 366) and the controls (*n* = 1, 347). This study covered 35.7% of all ELBW live births in Japan, including 2,382 and 2,421 cases in 2001 and 2002, respectively. Obstetrical complications were defined according to our previous reports [7]. Clinical chorioamnionitis was defined as a maternal temperature more than 38°C and at least one of the following four criteria: maternal tachycardia more than 100 bpm/min, uterine tenderness, white blood cell count more than 15,000, and foul smelling vaginal discharge. Fetal growth restriction (FGR) was defined as that with estimated weight less than 10th percentile. Oligohydramnios was diagnosed when the amniotic fluid index (AFI) was 8.0 or less or maximal fluid volume was 2.0 or less. Nonreassuring fetal status (NRFS) was defined as case with one or more of the following abnormal fetal heart rate patterns, including loss of fetal heart rate variability, recurrent late decelerations, recurrent severe variable decelerations, prolonged deceleration, or baseline fetal heart rate less than 120 beats per minute (bpm) or more than 160 bpm.

### 2.3. Statistical Analysis

Statistical analyses were performed using SAS 9.1 (SAS Institute, Cary, NC, USA). Our choice of risk factors for inclusion in the regression model was based on the results of univariable analysis. A relative risk with 95% confidence intervals (CI) was derived from these models to quantify the association between the causative determinant and obstetric complications. Unconditional logistic regression was used for multivariable analysis. The results were expressed as mean ± standard deviation (SD). Statistical analysis was carried out using the chi-square test, Fisher's exact probability test, and Wilcoxon signed-rank test. A *P* value of less than 0.05 was considered to be significant. Odds ratios (OR) with 95% CI were calculated to estimate the relative risk between the case and the control subjects with regard to the risk factors for neonatal death. Results were compared by both univariable and multivariable analysis. Logistic regression models were used to assess the influence of confounding factors. Survival rates according to birth weight or gestational weeks at delivery in cesarean delivery and in vaginal delivery were analyzed.

## 3. Results

### 3.1. Clinical Characteristics of Death and Survival Groups

Distribution of delivery weeks in this studied population is shown in Table 1. A comparison of the clinical characteristics in the death and survival groups is shown in Table 2. The risk factors rate of nonvertex presentation (48.1%), rate of vaginal delivery (63.9%), and incident rate of placental abruption (10.1%) were significantly higher in the death group as compared with the survival group. However, the risk factors rate of nonreassuring fetal status (30.9%), rate of premature rupture of the membranes (21.3%), rate of antenatal steroid administration (7.1%), birth weight (631.3 ± 152.3 g), gestational weeks at delivery (26.0 ± 2.7), and Apgar score at 5 minutes (1.8 ± 2.8) were significantly lower in the death group as compared with the survival group. In terms of the prevalence of maternal complications, such as placental abruption, placental previa, oligohydramnios, clinical chorioamnionitis, and FGR, there were no differences between the two groups. Cesarean sections were indicated for nonreassuring fetal status, nonvertex presentation, and previous hysterotomy.

### 3.2. Survival Rate of ELBW Infants

**Figure 1:**
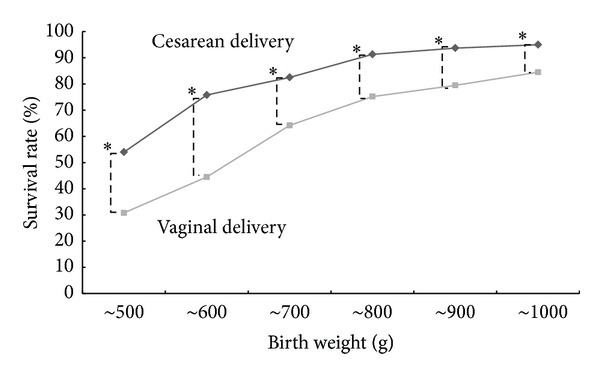
Survival rates for cesarean delivery were significantly higher than those for vaginal delivery at all birth weight levels in extremely low birth weight (400–1,000 g) infants. Asterisks indicate statistical significance (*P* < 0.05).

**Figure 2:**
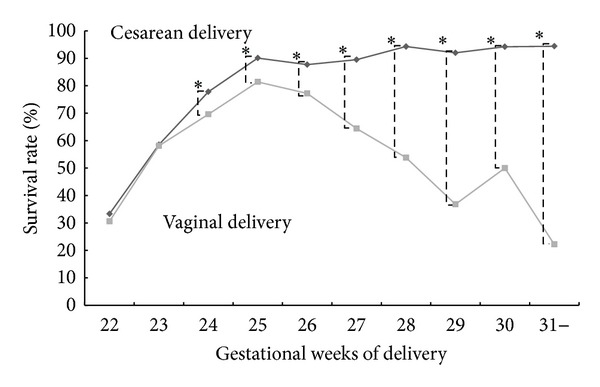
Survival rates for cesarean delivery were significantly higher than those for vaginal delivery from 24 to 31 gestational weeks of delivery in extremely low birth weight infants. Asterisks indicate statistical significance (*P* < 0.05).

**Figure 3:**
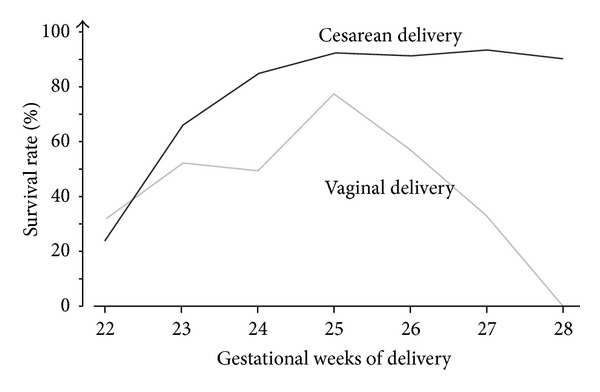
Survival rates according to delivery mode in nonvertex presentation of appropriate-for-date infants.

The total survival rate was 76.2% (366 versus 1,347). Survival rates by birth weight and by delivery method (vaginal or cesarean) are shown in Figure 1. Cesarean delivery showed a significantly higher survival rate at all birth weight levels as compared with vaginal delivery. Survival rates by birth weight at each gestational week of delivery from 22 weeks to 31 weeks are presented in Figure 2. Cesarean delivery showed significantly higher survival rates at 24 and 31 gestational weeks of delivery as compared with vaginal delivery, but there were no differences at 22 and 23 weeks. Survival rates according to delivery mode in nonvertex presentation of appropriate-for-date infants are shown in Figure 3.

### 3.3. Risk Factors for Survival in ELBW Infants (Table 3)

According to multiple regression analysis, nonvertex presentation (OR: 1.86, 95% CI [1.25, 2.76]), vaginal delivery (OR: 1.58, [1.04, 2.40]), and placental abruption (OR: 2.12, [1.06, 4.22]) were found to be significantly associated with neonatal death. However, the significant contributing factors for survival were antenatal steroid (OR: 0.41, [0.22, 0.75]), Apgar score at 5 minutes (OR: 0.56, [0.52, 0.61]), and birth weight (OR: 0.996, [0.995, 0.998]).

## 4. Discussion

The probability of survival of premature infants often depends on prematurity such as gestational age and birth weight [8]. However, many other factors are also at work such as race [9], level of neonatal care [1, 10], and presence of malformation [11]. Chromosomal abnormalities and malformation were excluded from the present study. In addition, this study was limited to data from Asians, who are considered to have the highest survival rates [12]. Moreover, the quality of neonatal care in Japan is high, with it being known to have the lowest neonatal mortality rate among countries included in the WHO database [6]. The present study includes data from all level III hospitals in Japan, where the quality of neonatal care is presumed to be the highest. Consequently, the results may more faithfully reflect factors that affect causes of mortality attributable to prematurity such as gestational age and birth weight. In reports published to date, survival rate and mortality rate have been investigated by week of gestation and by birth weight level, but VLBW infants have been included in the investigations when determining risk factors [13]. The cases in this study were limited to ELBW infants with a birth weight of less than 1,000 g. As a result, effects related to survival of VLBW infants weighing 1,000 g or more have been excluded. We concluded that this would allow us to derive certain important results from the standpoint of coming up with measures to take when encountering this limit of viability. It is assumed that the results of this study will serve as the answer to prematurity. That is, it is hoped that the present study will shed light on the current limit of viability.

The results of this study show that the overall survival rate in ELBW infants weighing 400–500 g is about 40%, and that in those weighing 500–600 g it exceeds 60%. In terms of week of gestation, the survival rate is 30% at 22 weeks and 60% at 23 weeks, and gestational age and birth weight with a survival rate that already exceeds 50% are 23 weeks and 500 g, respectively. In other words, it can be said that this is the 50% limit of viability. Moreover, the survival rate exceeds 80% beginning at 25 weeks of gestation. As shown in Figure 1, the survival rate in infants weighing 400–500 g delivered by cesarean section is about 1.8 times that for vaginal delivery or about 55%. However, the results in Figure 2 show no advantage for cesarean section at weeks 22 and 23 of gestation, with the difference becoming significant beginning at week 24. Therefore, cesarean section to save the life of the infant may be indicated in infants with an estimated body weight of ≥400 g or ≥24 weeks of gestation. Lee and Gould [14] conducted a large-scale retrospective study of VLBW infants and extraction methods in 2006. They reported that the survival rate in the caesarean section group improved significantly in infants weighing 500–700 g. Likewise, Malloy [15] retrospectively investigated the prognosis of VLBW infants in 2008. He reported that cesarean section at 22–25 weeks increased the survival rate of the infants. However, Batton et al. [16] reported that the overall mortality rate of infants did not improve significantly despite an increase in the number of cesarean sections over time, and they concluded that the use of cesarean section for premature infants should be questioned. However, the results of their investigations have demonstrated that the mortality rate is significantly lower in the caesarean section group when delivery is at 22 to 26 weeks of gestation. The problem becomes complicated if the long-term prognosis and neurological prognosis of neonates are considered, but if we limit the discussion to the short-term prognosis of neonates, the present study showed that cesarean delivery offered a clear advantage beginning at 24 weeks of gestation or a birth weight of ≥400 g. The present study did not shed light on the reason why cesarean section was beneficial to infants ≥24 weeks of gestation or weighing ≥400 g. However, it is presumed that vaginal delivery can be harmful to infants weighing less than 1000 g.

In terms of the incidence of maternal illness in the survival group and death group, the frequency of nonvertex presentation [17], vaginal delivery [14], and placental abruption [14, 18], which can generally be risk factors, was also significantly high in this study in the death group. However, it is very interesting that the occurrence of preterm rupture of membranes (PROM) and nonreassuring fetal status (NRFS) was significantly high in the survival group. This result might be more a problem in terms of care in the perinatal period. Earlier termination might have been chosen upon consideration of the fact that NRFS is a sign of placental factor and preterm PROM is a sign of intrauterine infection. This is supported by the results of multivariable analysis. In other words, while the odds ratios of nonvertex presentation, vaginal delivery, and placental abruption, which were determined to be poor prognostic factors in the survival group, all increased significantly and were greater than 1 according to the results of multivariable analysis, the odds ratios for NRFS and preterm PROM were not significant, but they were below 1 and were determined not to be risk factors. As reported by other researchers, antenatal corticosteroid administration reliably increases the viability of infants [2, 19]. There are not likely any who would voice a dissenting opinion to this. On the other hand, the occurrence of pregnancy induced hypertension (PIH), which is often seen in other reports [20, 21], was hardly seen at all and was not a risk factor. This was probably because the cases included in the present study were infants delivered in the second trimester, that is, weeks 26-27 of gestation, unlike other reports to date, because this study was limited to ELBW infants weighing less than 1,000 g. PIH generally occurs at weeks 32–34 of gestation. About 95% of the cases in this study, however, were less than week 32 of gestation, likely explaining why PIH was not a risk factor.

Placental abruption was a poor prognostic factor. The odds ratio was high, that is, 1.91, according to univariable analysis, and the odds ratio was also high, that is, 2.50, according to multivariable analysis. Signs of separation of the placenta, such as placental abruption and severe hemorrhage at parturition, have been identified as risk factors in reports published to date [14, 18, 21]. The cause of this has been identified as intrauterine infection. Ananth et al. retrospectively investigated the causes of placental abruption [22]. They reported that [1] an acute process, for example, intrauterine infection, and [2] a chronic process associated with fetal growth restriction and PIH acted alone or together to cause placental abruption. In their investigation of placental abruption at 20–36 weeks [2], was found to be four times more frequent than [1] overall. In terms of the timing of onset of placental abruption, onset at week 24 of gestation was the most frequent (6%) of the entire gestational period, and they found that the occurrence of placental abruption associated with an acute process was about 10 times more frequent as compared with full-term pregnancy. In addition, they found that PROM was a risk factor for acute placental abruption. The results of our present study showed that there was no difference in the incidence of fetal growth restriction and PIH, suggesting that the principal cause of placental abruption was an acute process, not a chronic process. Therefore, it is important to pay adequate attention to onset of placental abruption during delivery of ELBW infants. Since intrauterine infection markers allow detection of intrauterine infection before chorioamnionitis becomes clinically evident [23–25], it might be wise to consider them during delivery of ELBW infants. However, further studies on which delivery method to select when intrauterine infection is suspected are needed.

There are some limitations with respect to the accuracy or the depth in the clinical information due to the nature of a retrospective case-control study. However, we need to recognize that cesarean section is advantageous for ELBW infants. We are convinced of the need for further study by means of a well-organized and prospectively planned study.

In conclusion, in the present study, in which we did our best to remove risk factors other than those related to prematurity that affect the viability of premature infants, ELBW infants with a 50% survival rate were those at week 23 of gestation or with a birth weight of 500 g, and the present results suggested that cesarean section might improve viability beginning at week 24 of gestation or a birth weight of ≥400 g. Since nonvertex presentation, vaginal delivery, and placental abruption are poor prognostic factors in neonates, when performing vaginal delivery of ELBW infants at week 24 of gestation or later or with an estimated body weight of ≥400 g, it should be contingent on a vertex delivery, and measures may need to be taken to prevent placental abruption due to intrauterine infection.

## Figures and Tables

**Table 1:** Distribution of delivery weeks in this studied population.

Gestational weeks	Cesarean	Vaginal	Total
22	11	63	74
23	37	121	158
24	111	131	242
25	158	116	274
26	184	95	279
27	168	62	230
28	122	27	149
29	86	20	106
30	68	7	75
31	57	11	68
32	20	4	24
33	14	3	17
34	13	0	13
35	2	0	2
36	0	2	2

Total	1051	662	1713

**Table 2:** Comparison of clinical characteristics in the death and survival groups.

Putative risk factors	Death (*n* = 366)	Survival (*n* = 1,347)
Clinical chorioamnionitis	47 (12.8%)	217 (16.1%)
Premature rupture of the membranes	78 (21.3%)*	412 (30.6%)
Placenta previa	10 (2.7%)	40 (3%)
Fetal growth restriction	122 (33.3%)	448 (33.3%)
Oligohydramnios	43 (11.7%)	186 (13.8%)
Placental abruption	37 (10.1%)^†^	58 (4.3%)
Antenatal steroid	26 (7.1%)^†^	333 (24.7%)
Nonvertex presentation	176 (48.1%)^†^	465 (34.5%)
Nonreassuring fetal status	113 (30.9%)*	550 (40.8%)
Gestational week at delivery	26.0 ± 2.7^‡^	26.9 ± 2.5
Vaginal delivery	234 (63.9%)	403 (29.9%)
Male	169 (46.2%)	684 (50.8%)
Birth weight (gram)	631.3 ± 152.3^‡^	772.2 ± 148.2
Apgar score at 5 minutes	1.8 ± 2.8^‡^	6.7 ± 2.0

Data are presented as number (%) or mean ± SD. *Significant decrease according to chi-square test. ^†^Significant increase according to chi-square test. ^‡^Significant decrease according to Wilcoxon signed-rank test.

**Table 3:** Results of univariable and multivariable logistic regression analysis in terms of risk factors for neonatal deaths in extremely low birth weight infants.

	Univariable	Multivariable
Clinical chorioamnionitis	0.81 [0.61, 1.06]	0.88 [0.51, 1.52]
Premature rupture of the membranes	0.67 [0.54, 0.85]	0.81 [0.51, 1.30]
Placenta previa	0.94 [0.53, 1.64]	1.10 [0.34, 3.54]
Fetal growth restriction	1.00 [0.83, 1.22]	1.01 [0.85, 1.29]
Oligohydramnios	0.86 [0.65, 1.15]	1.36 [0.29, 4.29]
Placental abruption	1.91 [1.46, 2.51]*	2.50 [1.24, 5.03]*
Antenatal steroid	0.29 [0.19, 0.42]*	0.41 [0.22, 0.75]*
Nonvertex presentation	1.55 [1.29, 1.86]*	1.81 [1.21, 2.71]*
Nonreassuring fetal status	0.96 [0.77, 1.19]	0.82 [0.54, 1.24]
Gestational week at delivery	0.80 [0.76, 0.85]*	0.97 [0.87, 1.08]
Vaginal delivery	2.99 [2.48, 3.62]*	1.56 [1.00, 2.43]*
Male	0.86 [0.72, 1.04]	0.88 [0.59, 1.30]
Birth weight (100 grams)	0.994 [0.993, 0.995]*	0.996 [0.995, 0.998]*
Apgar score at 5 minutes	0.51 [0.48–0.55]*	0.56 [0.52–0.61]*

Values are presented as odds ratios [95% confidence intervals]. *Significant factors in predicting neonatal death. Underlines indicate significant data and bold indicates significant contributing factors for neonatal death.
